# The Clinical Value of Poor-Law Infirmaries

**Published:** 1915-02-20

**Authors:** H. W. Bruce

**Affiliations:** Medical Superintendent. The Infirmary, East Dulwich Grove.


*To the Editor of* The Hospital.

February 15, 1915.

### The Clinical Value of Poor-Law Infirmaries.

*To the Editor of* The Hospital.

Sir,—The fact that apparently the great majority of the medical superintendents of London infirmaries have made no reply to your article on clinical notes, etc., must not lead to the presumption that they agree that the statements made in it are true or the opinions expressed sound. To my mind it contained so many false generalisations and showed such ignorance and such unsoundness that it was useless to reply to it. It will doubtless do a little harm to the infirmaries. It was anonymous, and published as if with the authority of your paper, and it is certainly hard that such a type of journalism should be added to all that we have to put up with.—Yours faithfully,

H. W. Bruce, Medical Superintendent.

The Infirmary, East Dulwich Grove.
February 15, 1915.

[The article referred to and the letter published last week by the writer of the article gave the views of a medical man with Poor-Law experience, but not devoted wholly to the Poor-Law service. Its interest lay in that fact. Another article on the subject, by "A Former Superintendent," appears this week. Our own view will be expressed editorially later.—Ed. The Hospital.]

Medical Superintendent.

